# Downstream of GA_4_, *PbCYP78A6* participates in regulating cell cycle-related genes and parthenogenesis in pear (*Pyrus bretshneideri* Rehd.)

**DOI:** 10.1186/s12870-021-03098-z

**Published:** 2021-06-24

**Authors:** Haiqi Zhang, Wei Han, Huibin Wang, Liu Cong, Rui Zhai, Chengquan Yang, Zhigang Wang, Lingfei Xu

**Affiliations:** State Key Laboratory of Crop Stress Biology for Arid Areas, College of Horticulture, Northwest A&F University, Shaanxi Province, Taicheng Road No.3, Yangling, 712100 China

**Keywords:** Parthenocarpy, Pear, GA_4_, *PbCYP78A6*, Fruit development

## Abstract

**Background:**

Parthenocarpy results in traits attractive to both consumers and breeders, and it overcomes the obstacle of self-incompatibility in the fruit set of horticultural crops, including pear (*Pyrus bretshneider*). However, there is limited knowledge regarding the genetic and molecular mechanisms that regulate parthenogenesis.

**Results:**

Here, in a transcriptional comparison between pollination-dependent fruit and GA_4_-induced parthenocarpy, *PbCYP78A6* was identified and proposed as a candidate gene involved in parthenocarpy. *PbCYP78A6* is similar to *Arabidopsis thaliana* CYP78A6 and highly expressed in pear hypanthia. The increased *PbCYP78A6* expression, as assessed by RT-qPCR, was induced by pollination and GA_4_ exposure. The ectopic overexpression of *PbCYP78A6* contributed to parthenocarpic fruit production in tomato. The *PbCYP78A6* expression coincided with fertilized and parthenocarpic fruitlets development and the expression of fruit development-related genes as assessed by cytological observations and RT-qPCR, respectively. *PbCYP78A6* RNA interference and overexpression in pear calli revealed that the gene is an upstream regulator of specific fruit development-related genes in pear.

**Conclusions:**

Our findings indicate that *PbCYP78A6* plays a critical role in fruit formation and provide insights into controlling parthenocarpy.

**Supplementary Information:**

The online version contains supplementary material available at 10.1186/s12870-021-03098-z.

## Background

Angiosperms have evolved double-fertilization processes, which require coordinated communication between gametophytic and sporophytic tissues, and fruit development as pivotal steps of their survival and dispersal strategies [1, 2]. Fruit initiation requires successful pollination and fertilization, but parthenocarpy uncouples this synchronized association and triggers fruit development [3]. Phytohormones are thought to be triggers induced by fertilization. Many strategies to producing virgin fruit include the exogenous application or overproduction of plant hormones, particularly auxin and gibberellins (GAs) [4–6], as well as the mutation of specific genes in these two plant hormone signaling pathways [7, 8]. Auxin partially acts upstream of GAs in inducing fruit set [9]. Complex mechanisms involving different hormones have been revealed, but there is limited knowledge regarding the mechanisms that underlie parthenocarpy.

Parthenocarpy possesses traits attractive to both consumers and breeders. It also overcomes the obstacle of self-incompatibility in the fruit set of horticultural crops, including pear (*Pyrus bretschneideri* Rehd.) [3]. The fleshy fruit of pear is derived from the hypanthium and known as an accessory fruit. Owing to the traits of perennial fruit trees, knowledge regarding the mechanism behind parthenocarpy in pear is still limited. In recently years, research has mainly focused on exogenous applications of plant hormones to induce parthenocarpy in pear [10–14], whereas the genetic mechanisms underlying parthenocarpy are rarely studied.

Overexpressing CYP78A9, a member of cytochrome P450 78A subfamily, identified by a transfer DNA activation-tagging screen, produces the parthenocarpic phenotype in *Arabidopsis thaliana* [15]. AtCYP78A6 acts redundantly with AtCYP78A9 to control reproductive organ development [16]. To date, the catalytic function of CYP78A enzymes remain largely unknown, but the expression patterns and effects of related genes have been widely elucidated [15, 17, 18]. Overexpressing members of the CYP78A family increases non-autonomous cell proliferation and the sequential formatting of large organs [19, 20]. The growth of multicellular organisms are controlled by cell-cycle progression, which is mediated by the periodic activation of complexes containing cyclins (CYCs) and cyclin-dependent kinases (CDKs) [21]. The functions of CYP78A family members and their roles in parthenocarpy have not been reported, nor have correlations between their expression levels and cell proliferation in pear.

In this work, we provide a detailed description of *PbCYP78A6*’s functions in pear parthenogenesis. We produced matured fruits from pollination-dependent and GA_4_-induced ‘Dangshansu’ parthenocarpic pear. Using comparative transcriptome and qRT-PCR analyses, we determined that *PbCYP78A6* expression was intimately correlated with fruit set and development. Stable transgenes in tomato demonstrated the contribution of *PbCYP78A6* to parthenogenesis. The overexpression and RNA interference of *PbCYP78A6* in pear calli revealed that the gene is an upstream activator of cell proliferation. Thus, *PbCYP78A6*, induced by GA_4_, regulates parthenogenesis and cell proliferation in pear.

## Results

### A transcription analysis identified a cytochrome P450 gene, *PbCYP78A6*, potentially responsible for parthenocarpy

The GA_4_ treatments resulted in a relatively high fruit set rate of 93.2% and induced seedless fruits with decreased weights and increased fruit indices and hardness levels compared with seeded fruits, whereas GA_3_ treatments induced a relatively low fruit set rate of 56.8% and did not induce mature fruit formation (Table 1; Additional file 1: Fig. S1).

**Table 1:** The effects of GA_4_ and GA_3_ on ‘Dangshansu’ pear fruit set

Treatment	UP	HP	GA_4_	GA_3_
Fruit set rate	0d	87.9 ± 2.2b	93.2 ± 2.4a	47.0 ± 1.6c

Significant differences among treatments as determined by one-way ANOVA (*P* < 0.05) are indicated using different lowercase letters (a, b, c, d). The results represented are means of three biological replicates (± SD, *n* = 3)

*UP* Un-pollinated, *HP* Hand pollinated, *GA*_*4*_ GA_4_ treatments, *GA*_*3*_ GA_3_ treatments

**Fig. 1:**
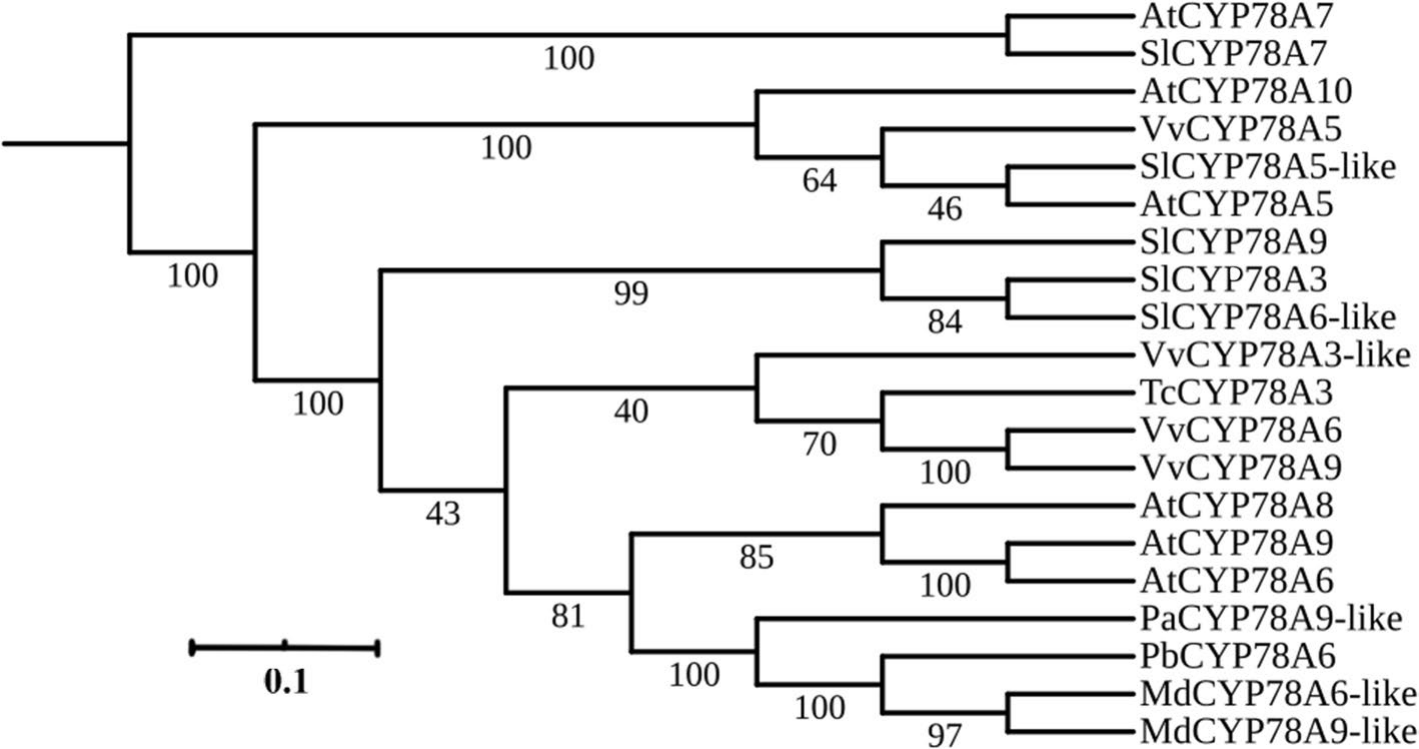
Phylogenic analysis of the PbCYP78A6 protein and CYP78A orthologs from other plant species. The protein accessions used were as follows: AtCYP78A7*(Arabidopsis thaliana*, NP_196559.1), SlCYP78A7 (*Solanum lycopersicum*, XP_004252635.1), AtCYP78A10 (*Arabidopsis thaliana*, NP_177551.1), VvCYP78A5 (*Vitis vinifera*, XP_002265310.1), SlCYP78A5-like (*Solanum lycopersicum*, XP_004236064.1), AtCYP78A5 (*Arabidopsis thaliana*, NP_172827.1), SlCYP78A9 (*Solanum lycopersicum*, XP_004240114.1), SlCYP78A3 (*Solanum lycopersicum*, XP_004230013.1), SlCYP78A6-like (*Solanum lycopersicum*, XP_004248458.1), VvCYP78A3-like (*Vitis vinifera*, XP_002266493.1), TcCYP78A3 (*Theobroma cacao*, XP_017973443.1), VvCYP78A6 (*Vitis vinifera*, RVW14892.1), VvCYP78A9 (*Vitis vinifera*, RVW91651.1), AtCYP78A8 (*Arabidopsis thaliana*, NP_171627.2), AtCYP78A9 (NP_191747.1), AtCYP78A6 (*Arabidopsis thaliana*, NP_182189.1), PaCYP78A9-like (*Prunus avium*, XP_021815024.1), PbCYP78A6 (*Pyrus bretschneideri*, XP_009375445.2), MdCYP78A6-like (*Malus domestica*, XP_008343443.2), MdCYP78A9-like (*Malus domestica*, XP_008343443.1). MEGA (version 5.10) was used to construct the phylogenetic tree with the Neighbor-joining method (1,000 replications bootstrap test and JTT model distribution)

**Fig. 2:**
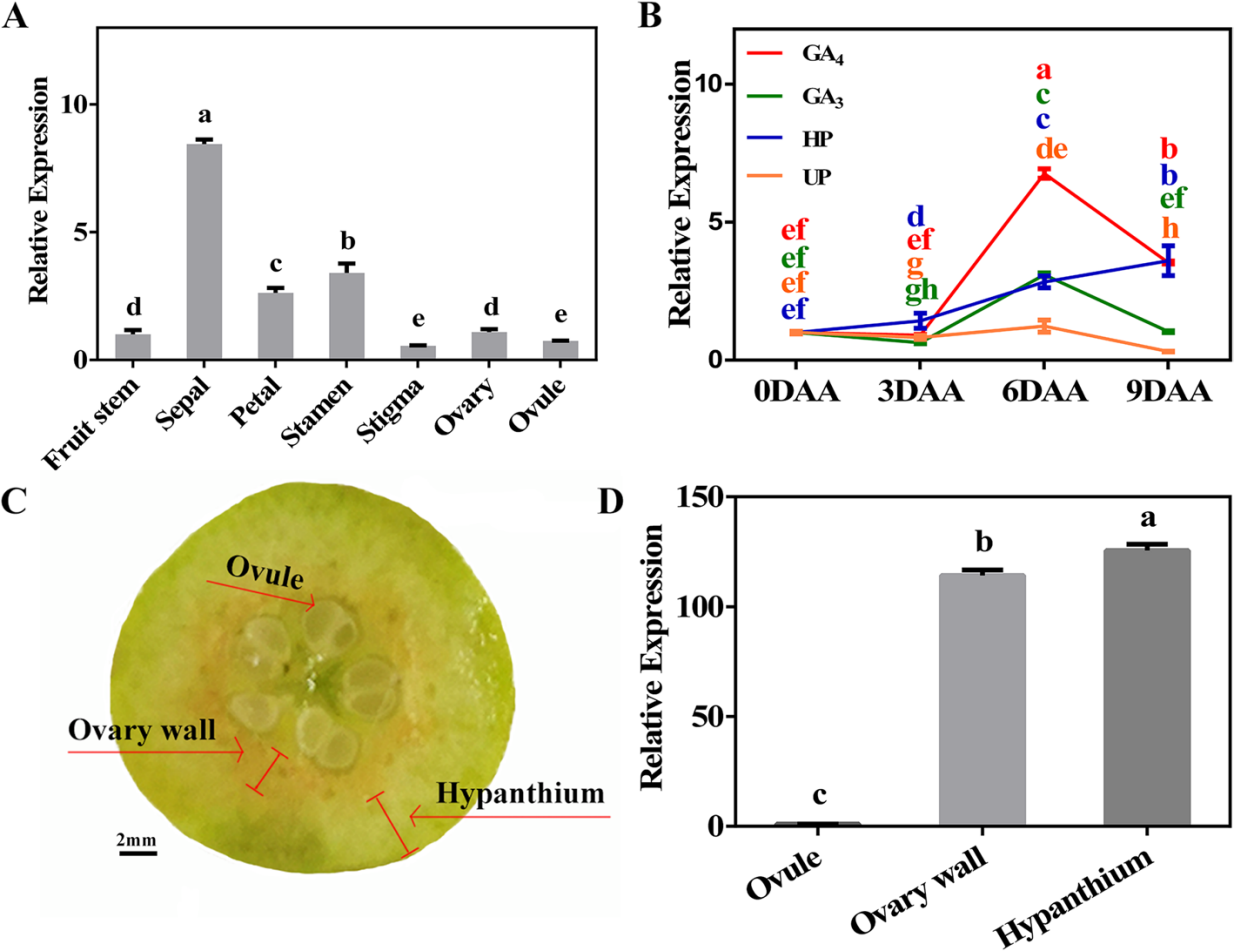
The *PbCYP78A6* transcript levels in fruitlets at distinct developmental stages and in different tissues of ‘Dangshansu’ pear. **A** The relative *PbCYP78A6* expression levels were detected in reproductive tissues of 3-day-old pear flowers; **B** The *PbCYP78A6* expression patterns in ‘Dangshansu’ fruitlets at early distinct stages; **C** Specific organs were sampled independently to detect the relative expression of *PbCYP78A6* in 30-day-old pear fruitlets; **D** *PbCYP78A6* transcript levels in different tissues of ‘Dangshansu’ fruitlets. An RT-qPCR analysis was used to determine the *PbCYP78A6* transcript levels. UP: Un-pollinated; HP: Hand pollinated; GA_4_: treated with 50 mg L^−1^ GA_4_; GA_3_: treated with 50 mg L^−1^ GA_3_. DAA: days after anthesis. The results are represented as means of three biological replicates (± SDs). Significant differences (*P* < 0.05) among treatments as determined by a one-way ANOVA are indicated using different lowercase letters

To further analyze the molecular mechanisms underlying parthenocarpy, an RNA-seq analysis was used to identify potential related genes. *PbCYP78A6* (LOC103964254) was commonly up-regulated in pollinated and GA_4_-treatment groups (Additional file 2: Fig. S2; Additional file 3: Supplementary Table S1). A phylogenetic analysis demonstrated that PbCYP78A6 in *P. bretshneider* displayed very high similarity levels to AtCYP78A9 and AtCYP78A6 (Fig. 1). *PbCYP78A6* was highly expressed in the sepal, which is an important component of pear fruit (Fig. 2A). During the early process of fruit set, *PbCYP78A6*’s expression level significantly increased in the effective treatment groups, including hand-pollinated and GA_4_-treated (Fig. 2B). We separated the developing fruit into hypanthium, ovary wall and ovule (Fig. 2C). A relatively high *PbCYP78A6* expression level was detected in the hypanthium compared with the ovary wall and ovule (Fig. 2D). We speculated that *PbCYP78A6* expression correlated with fruit development and parthenogenesis in pear.

### *PbCYP78A6* overexpression contributed to parthenocarpic fruit development in tomato

**Fig. 3:**
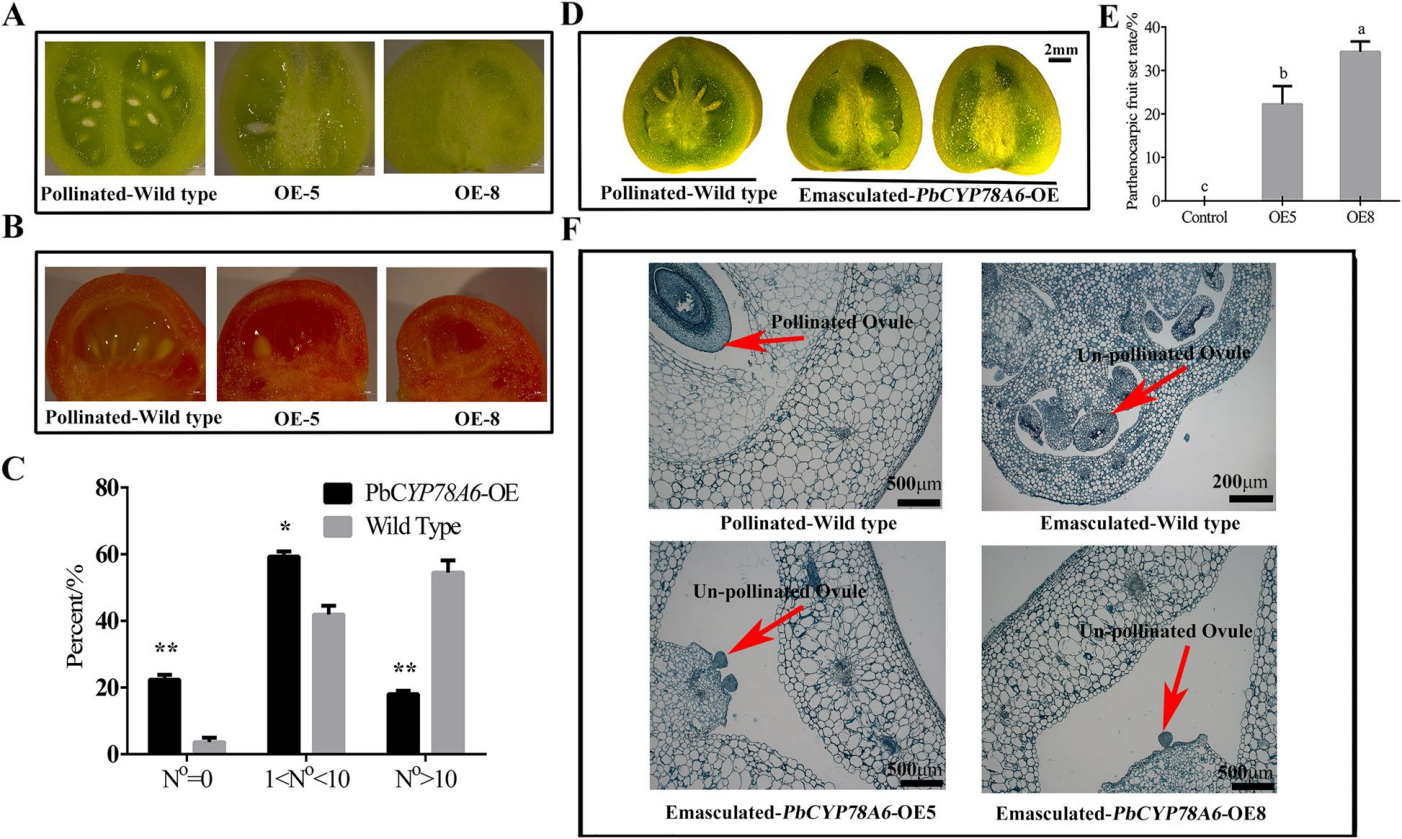
*PbCYP78A6* overexpression contributed to reduced seed numbers and parthenocarpy in tomato. **A** Longitudinal sections of wild-type and transgenic fruits produced by natural pollination at the green ripened stage; **B** Longitudinal sections of wild-type and transgenic fruits produced by natural pollination at the ripened stage; **C** The percentages of *PbCYP78A6-*overexpression (OE) fruits containing different numbers of seeds under natural pollination conditions; **D** Seeded fruit produced by hand pollination, and parthenocarpic fruit resulting from the emasculation of *PbCYP78A6-*OE lines; **E** The parthenocarpic fruit set rates of emasculated transgenic *PbCYP78A6-*OE tomatoes; **F** Histological observations of hand-pollination emasculated and emasculated transgenic *PbCYP78A6-*OE tomato pericarps at about 10 days after full-bloom. Bars = 200 μm in emasculated wild-type, other bars = 500 μm in **F**. The results represented are means of three biological replicates (± SDs). Significant differences (*P* < 0.05) among treatments as determined by a one-way ANOVA are indicated using different lowercase letters. Bars = 2 mm in **A** and **B**

To determine the potential roles of *PbCYP78A6* in fruit development and parthenogenesis, transgenic tomato lines overexpressing the *PbCYP78A6* gene were obtained (Additional file 4: Fig. S3A). Under natural pollination conditions, transgenic tomato overexpressing *PbCYP78A6* had significantly reduced numbers of seeds per fruit, and most of the fruits produced were seedless (Fig. 3A). At the mature stage, there were a few large seeds along with tracks of underdeveloped seeds in the transgenic fruits (Fig. 3B). Tomato lines with *PbCYP78A6* overexpression produced larger fruits than wild type lines (Additional file 4: Fig. S3B). Statistical analysis of seeds determined that overexpressing *PbCYP78A6* reduced the number of seeds per fruit (Fig. 3C). Seeded fruits were produced by pollinated ovaries in wild-type (WT) lines (Fig. 3D). The ovaries of *PbCYP78A6*-overexpression (OE) lines are capable of parthenocarpic fruits after being emasculated (Fig. 3D, E), whereas WT lines did not produce fruits without pollination. Similar to pericarps of pollinated ovaries, pericarps of emasculated ovaries overexpressing *PbCYP78A6* underwent cell division and expansion far more than that of emasculated ovaries (Fig. 3F), cell layers number and pericarps thickness of which were slightly less than that of the pericarps of pollinated fruits and much more than that of the pericarps of emasculated ovaries (Additional file 4: Fig. S3). Thus, *PbCYP78A6* was involved in the regulation of fruit development and parthenocarpy.

### Cell division and expansion were induced by GA_4_ and pollination as *PbCYP78A6* expression increased

**Fig. 4:**
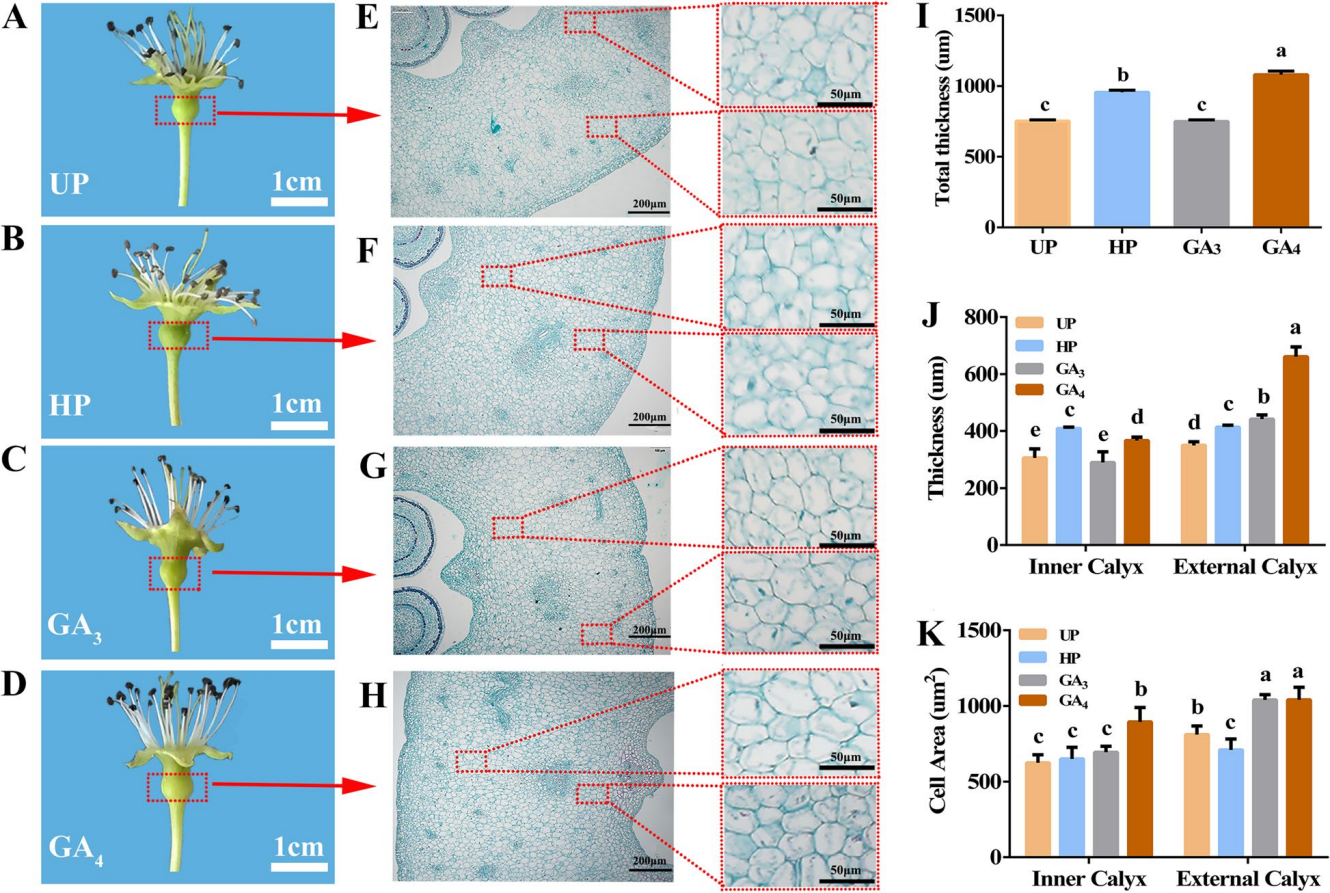
Phenotypic observations and histological features of ‘Dangshansu’ fruitlets 6 days after exposure to different treatments. **A**–**D** phenotypes of UP, HP, and GA_3_- and GA_4_-treated ‘Dangshansu’ fruitlets at 6 DAA, respectively; **E**–**H** histological observations of UP, HP, and GA_3_- and GA_4_-treated ‘Dangshansu’ fruitlets at 6 DAA, respectively; **I** quantification of the total thicknesses of the fleshy calyxes in treated ‘Dangshansu’ pear; **J** quantification of the internal and external thicknesses of the fleshy calyxes in treated ‘Dangshansu’ pear; **K** the cell areas of the internal and external fleshy calyxes in ‘Dangshansu’ pear. EC, External calyx (zone between the epidermis and the vascular bundle); IC, Internal calyx (zone inside the vascular bundle). Three fruitlets of each treatment were sampled for quantification. Error bars represent the standard deviation of the mean (± SDs; *n* = 12). Significant differences (*P* < 0.05) among treatments as determined by a one-way ANOVA are indicated using different lowercase letters

*CYP78A6* overexpression results in the production of large fruit owing to increased cell proliferation [15, 16]. To explore the cellular changes along with *PbCYP78A6* expression was highly induced, fruitlets were embedded in paraffin and sectioned. Phenotypic observations of the fruitlet sections were recorded (Fig. 4). The ovaries of the pollinated and GA_4_-treated groups were larger than those of the un-pollinated and GA_3_-treated groups (Fig. 4A–D). The thicknesses of the calyxes in GA_4_-treated and pollinated samples were significantly greater than those of the un-pollinated and GA_3_-treated groups (Fig. 4E–K). Compared with the un-pollinated treatment, hand pollination and GA_4_ exposure increased cell-layer production and cell-area enlargement (Fig. 4J, K). Thus, we speculated that *PbCYP78A6* might be a key regulator in the fertilized and parthenocarpic fruit growth.

### The expression of fruit development-related genes was activated during parthenogenesis

**Fig. 5:**
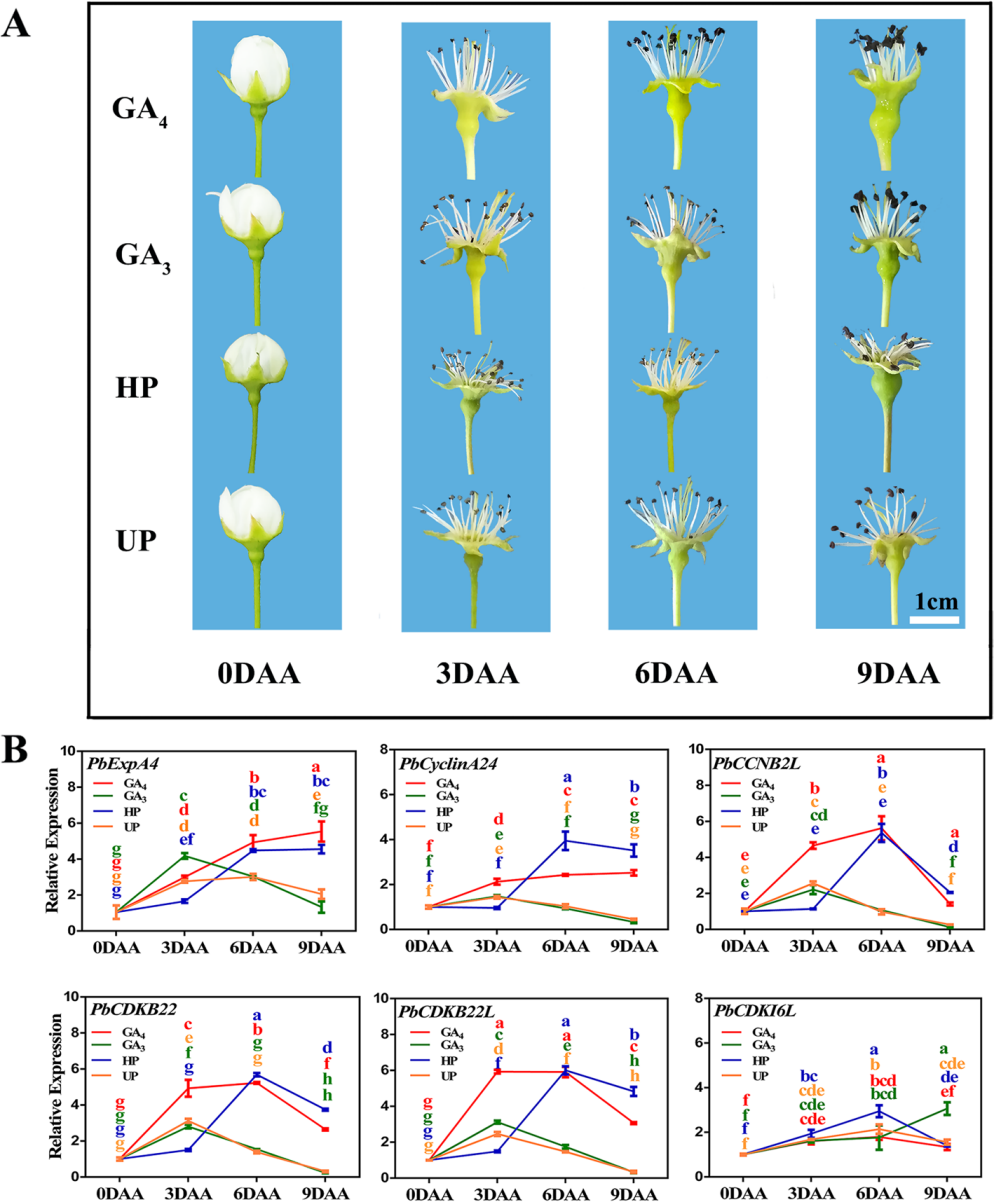
Dynamic growth of ‘Dangshansu’ fruitlets, and the transcript levels of cell cycle-related genes in ‘Dangshansu’ fruitlets at the early stage. **A** The dynamic growth of ‘Dangshansu’ fruitlets after UP, HP, GA_4_ and GA_3_ treatments at 0, 3, 6 and 9 DAA; **B** The expression levels of *expansin-A4* (*EXPA4*), *cyclinA2-4*; *G2/mitotic-specific cyclin-2-like* (*CCNB2L*), *cyclin-dependent kinase B2-2* (*CDKB22*), *Pbcyclin-dependent kinase B2-2-like* (*CDKB22L*) and *cyclin-dependent kinase inhibitor 6-like* (*CDKI6L*) in ‘Dangshansu’ fruitlets. The results represented are means of three biological replicates (± SDs). Significant differences (*P* < 0.05) among treatments as determined by a one-way ANOVA are indicated using different lowercase letters

To further understand the molecular mechanisms underlying parthenogenesis, the dynamic growth and correlated transcript levels of cell expansion- and division-related genes were observed in ‘Dangshansu’ fruitlets at an early stage. Notably, GA_4_-treated and pollinated fruitlets underwent rapidly growth, whereas GA_3_-treated and un-pollinated fruitlets did not show significant growth-related changes (Fig. 5A). On the basis of our previous studies [10–12], *expansin-A4* (*EXPA4*), *cyclinA2-4*, *G2/mitotic-specific cyclin-2-like* (*CCNB2L*), *cyclin-dependent kinase B2-2* (*CDKB22*), *Pbcyclin-dependent kinase B2-2-like* (*CDKB22L*) and *cyclin-dependent kinase inhibitor 6-like* (*CDKI6L*) were screened, and their expression levels played critical roles in fruit development. The RT-qPCR results showed that as the morphological changes occurred, the expression levels of these genes were significantly greater induced in pollinated and GA_4_-treated fruitlets than in un-pollinated and GA_3_-treated fruitlets (Fig. 5B). The *CDKI6L* expression level was repressed more by GA_4_ than GA_3_ (Fig. 5B). Intriguingly, the *PbCYP78A6* expression pattern was almost consistent with the those of fruitlet development-related genes (Figs. 2 and 5). We speculated that *PbCYP78A6* might act upstream of cell proliferation-related genes to regulate pear fruit growth.

### *PbCYP78A6* was involved in the regulation of fruit development-related genes

**Fig. 6:**
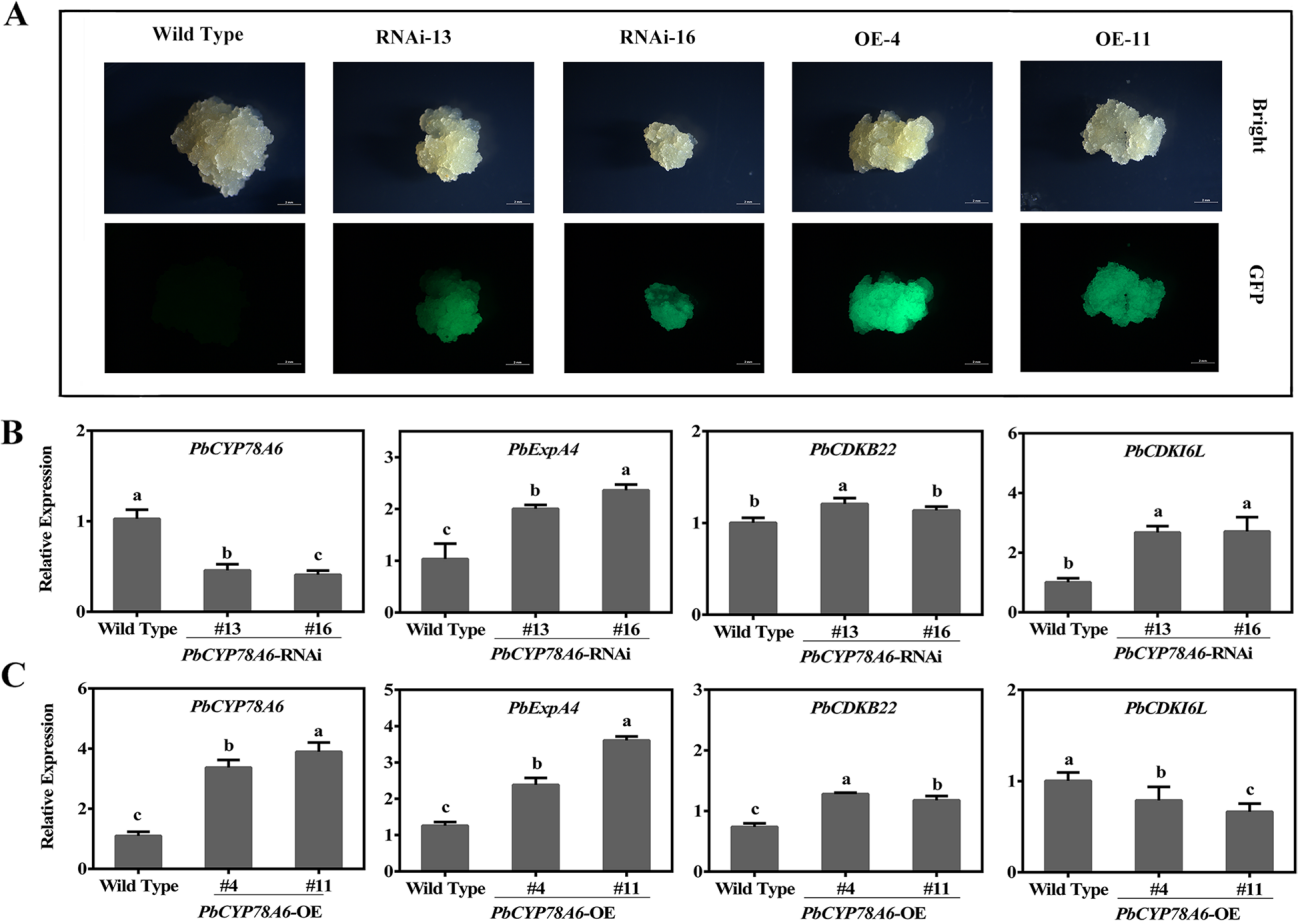
*PbCYP78A6* promotes the expression of cell cycle-related genes in pear calli. **A** Transgenic pear calli expressing green fluorescent protein (GFP) were detected. *PbCYP78A6*-RNAi, *PbCYP78A6* RNA interference; *PbCYP78A6*-OE, *PbCYP78A6* over-expression. **B** RT-qPCR analysis of *PbCYP78A6*, *PbEXPA4*, *PbCDKB22* and *PbCDKI6L* expression levels in *PbCYP78A6*-RNAi pear calli; **C** Quantitative RT-PCR analysis of the *PbCYP78A6*, *PbEXPA4*, *PbCDKB22* and *PbCDKI6L* expression levels in *PbCYP78A6*-OE pear calli. The results represented are means of three biological replicates (± SDs). Significant differences (*P* < 0.05) among treatments as determined by a one-way ANOVA are indicated using different lowercase letters

To further characterize the correlations between *PbCYP78A6* and parthenogenesis-related genes, transgenic *PbCYP78A6-*OE and -RNA interference (RNAi) calli were generated. Green fluorescent protein (GFP) signals were detected in *PbCYP78A6*-OE and -RNAi lines (Fig. 6A). RT-qPCR was used to confirm changes in *PbCYP78A6* expression (Fig. 6B, C). In *PbCYP78A6*-RNAi calli, *PbExpA4* and *PbCDKI6L* expression levels were drastically increased (Fig. 6B), and the *PbCDKB22*, *PbCDKB22L*, *PbCCNB2L* and *PbCyclinA24* transcript levels were not repressed (Fig. 6B; Additional file 5: Fig. S4), compared with in WT. However, in *PbCYP78A6*-OE calli, the positive regulators of cell proliferation, including *PbExpA4*, *PbCDKB22*, *PbCDKB22L* and *PbCyclinA24*, were significantly up-regulated, while a negative regulator of cell proliferation, *PbCDKI6L*, was down-regulated compared with that in WT (Fig. 6C; Additional file 5: Fig. S4). Thus, *PbCYP78A6* regulated cell proliferation by specific genes, which might be a key for fertilized and parthenocarpic fruit growth in pear.

## Discussion

In higher plants, an ovary can either be fertilized and subsequently grow into a fruit, or, owing to the lack of successful fertilization, it can enter the abscission process. Exogenous applications of multiple hormones mimic fertilization functions and fruit set, independently of fertilization, resulting in parthenocarpy [9, 22]. In ‘Dangshansu’ pear, GA_4_ is effective in inducing parthenocarpy [10–12, 23], whereas GA_3_ does not induce parthenocarpy. Here, the difference between GA_4_- and GA_3_-induction in pear was assessed to investigate the mechanisms of parthenocarpy-related downstream hormones.

In a comparison of transcriptomes between pollination-dependent and parthenocarpic fruit set, *PbCYP78A6* was identified as a candidate gene involed in the regulation of parthenogenesis. PbCYP78A6 is similar to AtCYP78A6 and AtCYP78A9 (Fig. 1), but its expression pattern differed from those of *AtCYP78A6* and *AtCYP78A9* [16, 20]. *PbCYP78A6* was determined to be widely expressed in reproductive organs, but it was highly expressed in the sepal (Fig. 2A), which partly develops into an accessory fruit in pear. *PbCYP78A6* expression was significantly activated by GA_4_ and pollination, and the expression level increased as the fruit set and developmental processes continued (Fig. 2B), which is consistent with *AtCYP78A9* activation being detected in developing seeds, septa, funiculi and placental tissues following fertilization [20]. Overexpressing *AtCYP78A6* or *AtCYP78A9* promotes the growth of not only seeds, but also the reproductive tissues, including sepals and siliques [16, 20], which indicates that *AtCYP78A6* and *AtCYP78A9* control their development. In pear, the cells of the exocarp are capable of cell division, in which new cell layers arise owing to periclinal cell division. In our study, *PbCYP78A6* expression was rarely detected in ovules, but occurred at a high level in the pericarp and hypanthium, in which cell division occurs vigorously (Fig. 2C, D). This indicated that *PbCYP78A6* may involve in pear fruit development. Thus, the different structures between fruit and silique hint at an important role for *PbCYP78A6* in parthenogenesis.

Under normal pollination conditions, the flowers of *PbCYP78A6*-OE transgenic plants produced fruits with reduced seed numbers and even some seedless fruits (Fig. 3A, B). This reproductive organ phenotype was similar to that seen in Arabidopsis overexpressing *AtCYP78A6* [16]. The auxin production and GA-mediated responses of ovules are dependent on pollination events that trigger fruit development [24]. Fruit development occurred normally in transgenic lines having low numbers of, and even no, seeds (Fig. 3A-C), which demonstrated that fruit generation occurred independently of the fertilization signals in *PbCYP78A6-*OE plants. Further emasculation experiments demonstrated that the flowers of transgenic plants had the capability of pollination-independent fruit development and producing parthenocarpic fruits (Fig. 3D-F). In Arabidopsis, large and seedless fruit have also been induced by the overexpression of CYP78A9 [15]. Here, we report that like *AtCYP78A9*, *PbCYP78A6* was capable of inducing parthenocarpic fruit set and development in tomato.

Silencing the Arabidopsis *CYP78A6/EOD3* homolog *PaCYP78A6* decreases fruit size by affecting cell proliferation [18], indicating that *PaCYP78A6* acts upstream of cell division and expansion. Here, the parthenocarpy produced by overexpressing *PbCYP78A6* maintains the activation of cell division and expansion similar to pollinated fruits (Fig. 3F). The differential expression of *PbCYP78A6* was consistent with the significantly increased cell division and expansion that occurred in the tissue-containing calyx and mature ovary compared with those of the un-pollinated group (Fig. 2). The process of fruit set is accompanied by cell division and expansion [25], indicating that *PbCYP78A6* plays an essential role in fruit formation.

Fruit development is largely dependent on cell division and expansion, and cell division is governed by two gene families, *CDK*s and *CYC*s [26]. Two other families, *EXPA* and *EXPB*, have the ability to regulate cell expansion by extending cell walls [27]. CDKBs, CYCAs and EXPAs participate in regulating fruit development [28–30]. In accordance with our previous study [10, 12], the expression levels of selected cell division- and expansion-related genes were analyzed during the early fruit set stage (Fig. 5). Among them, *PbExpA4*, *PbCyclinA2-4*, *PbCCNB2L*, *PbCDKB22* and *PbCDKB22-like* had up-regulated expression pattrens, which agreed with the histological observations (Fig. 4). Moreover, a CDK inhibitor gene, *CDKI6L*, was identified, and its expression decreased in GA_4_-treated fruitlets. However, the GA_3_ treatment failed to repress the expression of *CDKI6L*. ICK1 and ICK2 expression reduce the CDK activity and affect cell division in *A. thaliana* [31], indicating that the inhibition of CDKI6L also plays an important role in fruit development. Thus, these genes are important for pear fruit development. Intriguingly, *PbCYP78A6* expression was almost consistent with the expression patterns of *PbEXPA4*, *PbCyclinA2-4*, *PbCCNB2L*, *PbCDKB22* and *PbCDKB22-like*, and it was negatively correlated to the expression of the repressor *CDKI6L* (Figs. 2B and 4B).

**Fig. 7:**
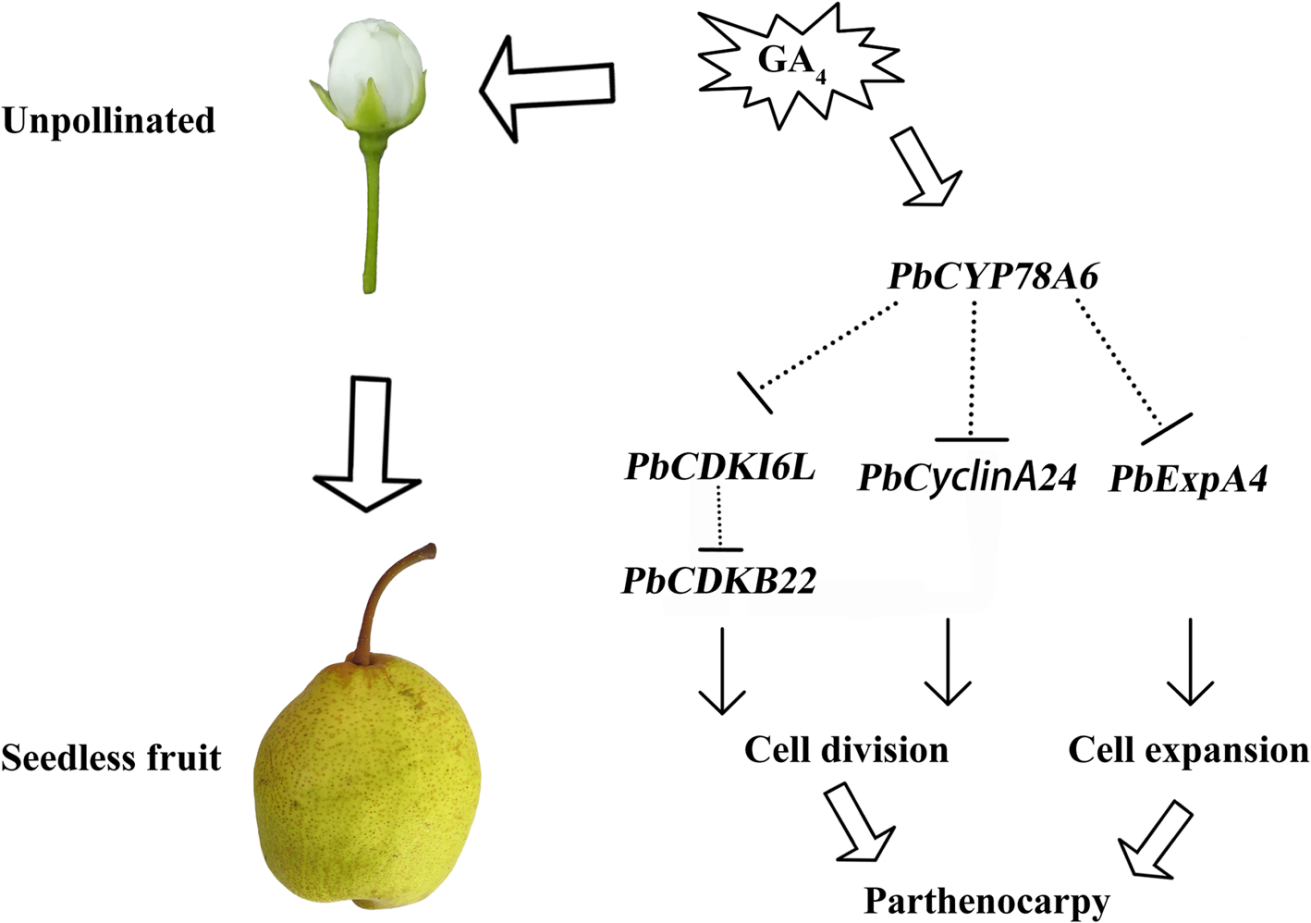
Model depicting the role of *PbCYP78A6* in controlling fruit development-related genes to regulate parthenocarpy

*PaCYP78A9*’s effect on plant organ size is regulated by cell cycle-related genes [18], whereas *PbCYP78A6*’s effect on fruit development might be mediated by specific fruit development-related genes in pear. In *PbCYP78A6*-RNAi pear calli, silencing *PbCYP78A6* did not halt cell proliferation (Fig. 6B; Additional file 5: Fig S4A). Overexpressing *PbCYP78A6* promoted the expression of fruit growth-related genes and particularly repressed *PbCDKI6L* expression in pear calli (Fig. 6C; Additional file 5: Fig S4B). The *PbCDKB22*, *PbCDKB22-like*, *PbCyclinA2-4* and *PbCCNB2L* expression levels were negatively correlated with the *PbCYP78A6* expression level. Similarly, silencing *PaCYP78A6* or *PaCYP78A9* does not completely repress the expression of all the cell proliferation-related genes [18, 32]. The evidence indicates that there might be other factors involved in controlling cell proliferation. We also identified a CDK repressor, *PbCDKI6L*, whose expression was up-regulated as *PbCYP78A6* expression decreased (Fig. 6). The reduced CDK activity was attributed to the increased ICK1 expression, which represses cell division [31]. Thus, *PbCDKI6L* plays a key role in the regulation of *PbCYP78A6* expression in fruit development. Cell-wall loosening is critical for rapid cell division, and it is most often controlled by EXPs [33]. Both silencing and overexpressing *PbCYP78A6* significantly promoted *PbExpA4* expression, perhaps owing to the consequences of cell proliferation. Although the effect of *PbCYP78A6* was confirmed on cell cycle related genes and parthenogenesis, the catalytic function of the PbCYP78A6, even CYP78A members in Arabidopsis, enzymes remains largely unknown. It is predicted that AtCYP78A9 is located in the flavonoid branch of the phenylpropanoid pathway [20]. There are no available predictions for AtCYP78A6. Characterization of metabolite differences between mutants in this gene family is tentatively carried out and perturbations in the flavonol biosynthesis pathway were detected in mutants which suggest a relationship between the phenylpropanoid pathway and parthenocarpy [20]. It is of interest that expression of several phenylpropanoid-related genes shows higher in the highly parthenocarpic group compared to weakly parthenocarpic pear cultivars [34]. It implies the engagement of PbCYP78A6 in the biosynthesis of some unknown type of plant growth regulator. The metabolite of PbCYP78A6 and the mechanism underlying its functions on the parthenocarpy remain to be further investigated. Our findings indicate that *PbCYP78A6* can regulate parthenogenesis and act as an upstream regulator of fruit development (Fig. 7).

## Conclusions

*PbCYP78A6* was induced by pollination and GA_4_ treatments, and its overexpression resulted in parthenocarpic tomato. The effects of *PbCYP78A6* on fruit development may be mediated by cell cycled-related genes. The utilization of CYP78A6 by fruit trees and its altered expression in fruits might provide a method for producing seedless fruits and enhancing crop yields (Fig. 7). The further elucidation of unknown plant growth-related substances may contribute to their utilization in regulating fruit development.

## Methods

### Plant material and growth conditions

Treated ‘Dangshansu’ pear trees (*Pyrus bretshneider* Rehd.) were planted in Pear experimental base of Northwest A&F University located in MeiXian County, Shaanxi Province, China (34.28°N, 108.22°E; 562 m). The average annual precipitation is 574.6 mm, and the average annual temperature was 12.7 °C. 16-year-old ‘Dangshansu’ pear trees grafted onto *Pyrus betulifolia* Bge rootstocks were used as the experimental materials. Permissions for all the materials used in this experience have been obtained.

Micro-Tom (*Solanum lycopersicum* L.) was chose for transformation in this experiment. Seeds were received from the gift of Xiangqiang Zhan, from Northwest A&F University, Yangling, China. For transgenic experiments, seeds were soaked with sterile water and sterilized with 2% Sodium hypochlorite solution. Sterilized seeds were germinated and grown in glass jars covered with transparent lips, containing 30 ml medium [Murashige and Skoog (1/2 MS) salts, 1.5% (w/v) sucrose, and 0.74% (w/v) agar], then put in a culturing chamber at 25 °C under 16-h/8-h light/dark conditions for 6–8 days until cotyledons were fully extended, which were used for next transformation experiments.

### Experimental treatments and samples

Two days before anthesis, all of the treatments and the control were bagged to avoid pollination. To avoid the disturbing fruit set caused by natural pollination, the opened flowers and the weak buds were removed to make sure every single pear flower keeping consistent and unopened when the exogenous hormones were applied. GA_3_ solutions 50 mg L^−1^ and GA_4_ solutions of 50 mg L^−1^ were independently sprayed on individual un-pollinated flowers of ‘Dangshansu’ pears at anthesis. 1:1 water: ethanol mixture was sprayed on un-pollinated flowers as the un-pollinated treatment. Three branches of each treatment were used as three replicates. Fruitlets with complete structure per treatment were randomly sampled. Fruits at 0, 3, 4, 6, 9, 30 and 145 DAA were harvested for morphological observation and other experiments, respectively. After removing the stalks, sepal, stamens an gynoecium, fruitlets left sampled at 4 and 6 days after anthesis (DAA) were used for RNA sequencing and immediately fixed in formaldehyde–acetic acid–alcohol for paraffin section, respectively. Fruitlets at 0, 3, 6, 9 DAA were used for the analysis of gene expression patterns with the same dispose as above. Fruitlets with hand pollination at 3 DAA were subdivided into fruit stem, sepal, petal, stamen, stigma, ovary, and ovule for tissue-specific expression. Fruits at 30 DAA were subdivided into pericarp, hypanthium, ovary wall and ovule for the expression position of *PbCYP78A6* in fruits. Each sample was harvested from 12 fruitlets, pooled, directly frozen in liquid nitrogen and stored at − 80 °C.

### Determination of fruit set rate

A total of 30 blooms on each three branches was labeled and bagged immediately after receiving treatments. At 20 DAA, the bags were removed. The formula used to calculate the fruit-set rate was as follows:

$$\text{Fruit}\hspace{0.25em}\mathrm{set}\hspace{0.25em}\left(\%\right)=\left(\text{number of fruitlets remaining}/\text{number of total flowers counting}\right)\times100.$$

### Paraffin sectioning and data statistics

To conduct the histological observations, fruit samples of four treatments in ‘Dangshansu’ pear were collected at 6 DAA when the significant changes were observed, immediately fixed in formaldehyde–acetic acid–alcohol fixative [35] and stored at 4 °C. The ovaries were dehydrated in an ethanol/xylene series and embedded in paraffin. They were then cut into 8-μm-thick slices, dried and stained with safranin and fast green. The anatomical images were observed using a microscopic imaging system (BX51 + PD72 + IX71, OLYMPUS, Japan). Cell area and calyx thickness were calculated using Image J software (https://imagej.net/Welcome), with three sections from three fruitlets used for each measurement. Cell area calculation method was as follows: first, a certain area was randomly circled and the total cell area was calculated; second, the total number of cells was counted; and third, the representative cell area of a single cell was calculated as the total cell area divided into the total number of cells. All each calculation was performed on more than three replicates.

### Transcription analysis

To further understand the potential molecular mechanism underlying parthenocarpy, Samples from unpollinated, hand pollinated, GA_3_-treated (without pollination) and GA_4_-treated (without pollination) at 4 DAA were utilized for RNA sequencing. Significant morphological changes happened from 3 to 6 DAA, so fruitlets at 4 DAA were selected to conduct RNA-seq experiment. Three independent biological replications were sequenced and analyzed. 12 samples (Un-pollination, Hand pollination, GA_3_ and GA_4_ treated samples of ‘Dangshansu’ pear at 4 DAA with three biological replicates, respectively) were subjected to total RNA extraction and Illumina HiSeq TM sequencing. Generating at least 40.13–62.70 million raw reads among each library was followed by filtering process. After then, 39.77–61.99 million clean reads remained with a Q30 percentage (an error probability lower than 0.1%) ranging from 90.77 to 95.17%. The clean reads accepted were mapped to the reference pear (*P. bretschneideri* Rehd.) genome by using HISAT software. Range from 72.35–80.15% of total reads were mapped to the reference genome (Additional file 6: Supplementary Table S2). Over 95.9% of reads mapped to genome were mapped to genomic exon regions. FPKM (expected number of Fragments Per Kilobase of transcript sequence per Millions base pairs sequenced) was used to evaluate genes expression level. FPKM > 1 was termed as standard to determine the expression of genes. 12 genes were selected to verify the reliability of transcription data by qRT-PCR (Additional file 7: Fig. S5). The RNA-Seq data of un-pollinated ovaries were used as the controls. padj < 0.05 and |log2 (ratio)|> 1 were used as the thresholds to determine the significance of DEGs. The DEGs were identified by pairwise comparisons of the 4 libraries, HP vs. UP, GA_4_ vs. GA_3_ in ‘Dangshansu’ pear. Pearson correlation between biological replicates ranged from 0.974–0.987. Gene was annotated using the ‘Dangshansuli’ (http://www.ncbi.nlm.nih.gov/genome/?term=pyrus) database as a reference. Venn diagram analysis was used to perform differential expression analysis. Kyoto Encyclopedia of Genes and Genomes (KEGG) functional annotations were based on sequence homologies against public database (www.genome.jp/kegg/). The expression profiles of DEGs were performed on TB-tools [36].

### Real-time quantitative PCR (RT-qPCR) validation of gene expression levels

The qRT-PCR was performed on a Step One plus Real-Time PCR Instrument (Thermo Fisher Scientific, Massachusetts, USA) machine using TB Green premix Ex *Taq* II Kit (Takara, Dalian, China). About 0.3 g samples were weighed and quickly grinded to powder in liquid nitrogen. Total RNA was extracted using a Polysaccharides and Polyphenolics-rich RNAprep Pure Plant kit (Tiangen, Beijing, China). RNA concentration and quality were assessed by spectrophotometry on Thermo Scientific Microplate Reader (multiscan GO) and Polypropylene gel electrophoresis on electrophoresis meter (DYY-6D), respectively. cDNA was synthesized by the reversely transcription of 1 μg total RNA using a PrimeScript RT reagent kit with gDNA Eraser (Takara, Dalian, China). Primers of target genes were designed by Primer Premier 5.0 software (PREMIER Biosoft) and NCBI Primer-BLAST tools (https://www.ncbi.nlm.nih.gov/tools/primer-blast/index.cgi). The Actin 7 gene was used as the internal reference for the gene expression analysis. Primers for verification of transcription data were described in Additional file 8: Supplementary Table S3. Primers for *PbCYP78A6* (LOC103964254), *Pbcyclin-dependent kinase B2-2* (LOC103961775), *Pbcyclin-dependent kinase B2-2like* (LOC103952922), *Pbexpansin-A4* (LOC103951053), *G2/mitotic-specific cyclin-2-like* (*CCNB2L*) (LOC103962422), *PbCyclinA2-4* (LOC103931294), *PbCDKI6-like* (LOC103964480) were listed in Additional file 8: Supplementary Table S3. The PCR reactions were carried out using an initial incubation at 95 °C for 30 s, and then 40 cycles of 95 °C for 5 s and 60 °C for 30 s. All reactions were performed on three biological and three technical replicates. Relative quantification of specific mRNA levels was performed using the cycle threshold (Ct) 2^−ΔΔCT^ method [37].

### Phylogenetic analysis

The full-length CDS of *PbCYP78A6* (LOC103964254) was isolated from ‘Dangshansu’ pear (http://www.ncbi.nlm.nih.gov/genome/?term = pyrus). Amino acid sequences of PbCYP78A6 and other CYP78A subgroup members of other plants were aligned using ClustalW [38]. MEGA5.10 was applied to construct the phylogenetic tree with the neighbor-joining statistical method. In addition, 1000 bootstrap replications were performed for testing of phylogeny [39].

### Construction of plasmids and generation of transgenic pear calli

To generate transgenic pear calli, the coding region of *PbCYP78A6* was cloned using primers *PbCYP78A6*-OE F/R and introduced into the vectors pGWB414 vector based on gateway recombination technology (Invitrogen) to create an overexpression vector. The vectors pHellsgate2 and pK7WIWG2D were used as RNAi-mediated vectors for silencing *PbCYP78A6* as described [40, 41]. The vectors were transformed into *Agrobacterium tumefaciens strain* EHA105 for infiltration.

The induction of pear calli and their transformation were described previously [42]. Briefly, the prefabricated suspension of EHA105 was incubated with 15-day-old pear calli for 15 min. After co-cultured on MS medium containing 1.0 mg·L^−1^ of 2, 4-D and 0.25 mg · L^−1^ of 6-BA for 2 days at 24 °C. Subsequently, the calli were washed three times with sterile water containing 300 mg·L^−1^ of Cefotaxime sodium and transferred to MS medium supplemented with 300 mg · L^−1^ of Cefotaxime and 50 mg·L^−1^ of kanamycin sulfate for transgene selection.

### Production of transgenic lines in tomato

The full-lenth *PbCYP78A6* (LOC103964254) coding sequence (CDs) was isolated from ‘Dangshansu’ cDNA using primers with adaptors (Additional file 9: Supplementary Table S4) designed by Snap Gene software 1.1.3, and then cloned into the *BamH I* and *SacI* restriction enzyme sites downstream of a *Cauliflower mosaic virus* (CaMV) 35S promoter in the pBI121 vector to generate a overexpression construct using a ClonExpress One Step Cloning kit (Vazyme, Nanjing, China). The recombinant vect was transformed into *Agrobacterium tumefaciens strain* LBA4404 by heat-shock method, and the positive monoclonal *Agrobacterium* cell proliferated at 28 °C, 200 rpm, dark in Luria–Bertani (LB) solid medium containing appropriate antibiotics (kanamycin and rifampicin). After incubation for appropriate time, the *Agrobacterium* cell concentration achieving to OD_600_ ≈0.5–0.8 were centrifuged at 600 rpm, collected in the tube, and then suspended in MS isopyknic to LB solution. The bacterial suspension was used for tomato transformation.

Transformation assays were carried out as previously described [43]. Briefly, sterilized tomato seeds were grown until its cotyledons full stretched in glass jars. Cotyledons were cut into sections and placed on Petri dishes containing solidified MS1 medium for 2 days in the dark, Then immersed them in bacterial suspensions prepared above for 10 min. Explants impregnated with bacterial suspension were blotted with filter paper, then cultured in the dark for 2 days in MS1 medium. Then the explants were transferred to MS2 solidified medium. After explants developed resistant calli produced shoots, 1–2 cm shoots were excised and placed on MS3 medium in glass jars. After took root, explants with root were cultured in pots containing vermiculite, watered with Hoagland’s solution, and conditioned in a growth chamber before transferring to the greenhouse. Progeny from the transgenic plants were obtained by selfing under controlled conditions.

Medium used above were as follow: Suspension liquid included MS salts supplemented with 0.4 mg l^−1^ thiamine hydrochloride, 100 mg l^−1^ myo-inositol, 2% (w/v) sucrose and 200 μM acetosyringone. MS1 contained MS salts supplemented with vitamins, 3% (w/v) sucrose, 100 mg l^–1^ myo-inositol, 4 mg l^–1^ indole acetic acid (IAA), 4 mg l^–1^ kinetin, and 0.8% (w/v) agar. MS2 contained MS1 supplemented with 1 mg l^–1^ zeatin, 300 mg l^–1^ cefatoxime, and 100 mg l^–1^ kanamycin. MS3 was consist of MS salts, 2% (w/v) sucrose, 100 mg l^–1^ myo-inositol, 1 mg l^–1^ thiamine, 0.1 mg l^–1^ IAA, and 0.8% (w/v) agar.

## Supplementary Information

**Additional file 1: Figure Supplemental 1.** Seeded fruits produced by fertilization and parthenocarpic fruits induced by GA_4_ in ‘Dangshansu’ pear.

**Additional file 2: Figure Supplemental 2.** Identification of *PbCYP78A6* with transcription analysis (A) Number of differentially expressed genes between hand pollination treatment and un-pollination, GA_4_-treatment and GA_3_-treatment using venn diagram. (B) Top 20 pathways of KEGG functional enrichment among common DEGs between HP vs. UP and GA_4_ vs. GA_3_. (C) Relative expression of genes with the absolute value of fold change (|log2|>1).

**Additional file 3: Supplementary Table S1.** Common differentially expressed genes in pollinated and GA4-treatment groups.

**Additional file 4: Figure Supplemental 3.** Detection of the selected transgenic lines with overexpressing *PbCYP78A6* gene and morphological, histological features of transgenic tomatoes. (A) The expression of *PbCYP78A6* gene in transgenic tomatoes. (B) The morphological features of tomato lines with PbCYP78A6 overexpression and wild-type lines at the same trusses position under natural pollination. (C) The cell layers of transgenic tomatoes ovaries pericarp in *PbCYP78A6* overexpression tomatoes. (D) The pericarp thickness of transgenic tomatoes ovaries in *PbCYP78A6* overexpression tomatoes. EW, Emasculated Wild-type; PW, Pollinated Wild-type. The results represented are means of three biological replicates (±SD). Significant differences (P< 0.05) among treatments as determined by One-way ANOVA are indicated using different lowercase letters.

**Additional file 5: Figure Supplemental 4.** The expression of cell division and expansion related genes in transgenic calli. A, RT-qPCR analysis of the expression levels of *PbCDKB22L*, *PbCCNB2L*, *PbCyclinA24*, in *PbCYP78A6* RNAi pear calli. B, RT-qPCR analysis of the expression levels of *PbCDKB22L*, *PbCCNB2L*, *PbCyclinA24* in *PbCYP78A6* OE pear calli. The results represented are means of three biological replicates (±SD). Significant differences (P< 0.05) among treatments as determined by One-way ANOVA are indicated using different lowercase letters.

**Additional file 6. **The percentages of total clean reads mapped to the reference pear (P. bretschneideri Rehd.) genome.

**Additional file 7: Figure Supplemental 5.** The FPKM and relative expression of genes were selected to determine the reliability of transcriptome data. The results represented are means of three biological replicates (±SD). Significant differences (P< 0.05) among treatments as determined by One-way ANOVA are indicated using different lowercase letters.

**Additional file 8: Supplementary Table S3.** List of qRT-PCR primers.

**Additional file 9: Supplementary Table S4.** List of primers cloning *PbCYP78A6-like*.

## Data Availability

Permissions for all the materials used in this experience had been obtained. No further permission was therefore needed. All data generated and analyzed during this study are included in this published article. Extra data has been appended as supplementary Tables. All the genes’ sequence and information can be accessed on National Center of Biotechnology Information (https://www.ncbi.nlm.nih.gov/).

## Abbreviations

GA_4_: Gibberellin A4
GA_3_: Gibberellin A3
HP: Hand pollination
UP: Un-pollination
OE: Over-expression
EXPA4: Expansin-A4
CCNB2L: G2/mitotic-specific cyclin-2-like
CDKB22: Cyclin-dependent kinase B2-2
CDKB22L: PbCyclin-dependent kinase B2-2-like
CDKI6L: Cyclin-dependent kinase inhibitor 6-like
RNAi: RNA interference
SD: Standard deviation
