# Supplementary figures and images for: Downstream of GA4, PbCYP78A6 participates in regulating cell cycle-related genes and parthenogenesis in pear (Pyrus bretshneideri Rehd.)
Source: BMC Plant Biol. 2021 Jun 24;21:292. doi: 10.1186/s12870-021-03098-z (PMC8223387; doi:10.1186/s12870-021-03098-z)

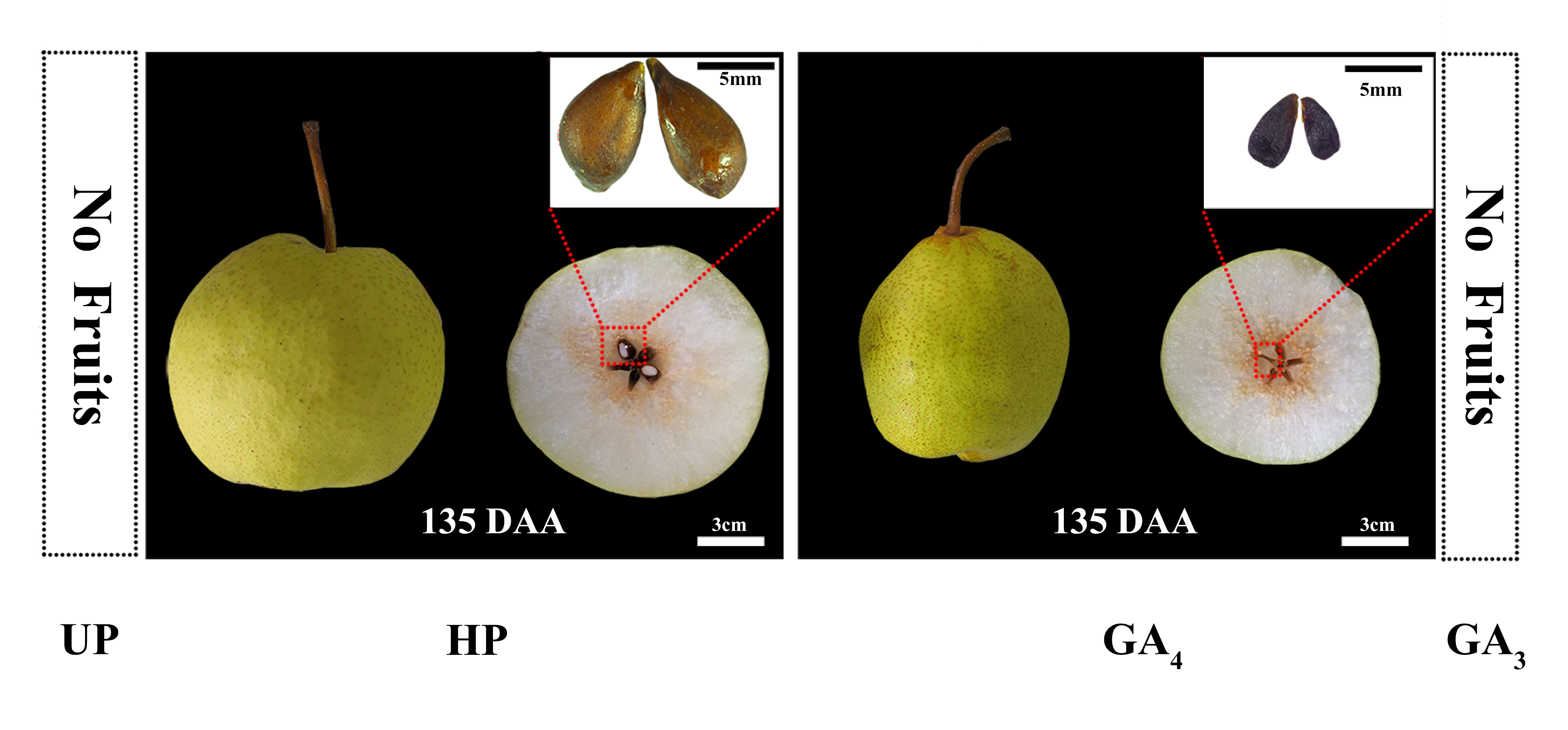

Supplement: Supplementary file 1 — Additional file 1: Figure Supplemental 1. Seeded fruits produced by fertilization and parthenocarpic fruits induced by GA4 in ‘Dangshansu’ pear. [file 12870_2021_3098_MOESM1_ESM.tif]

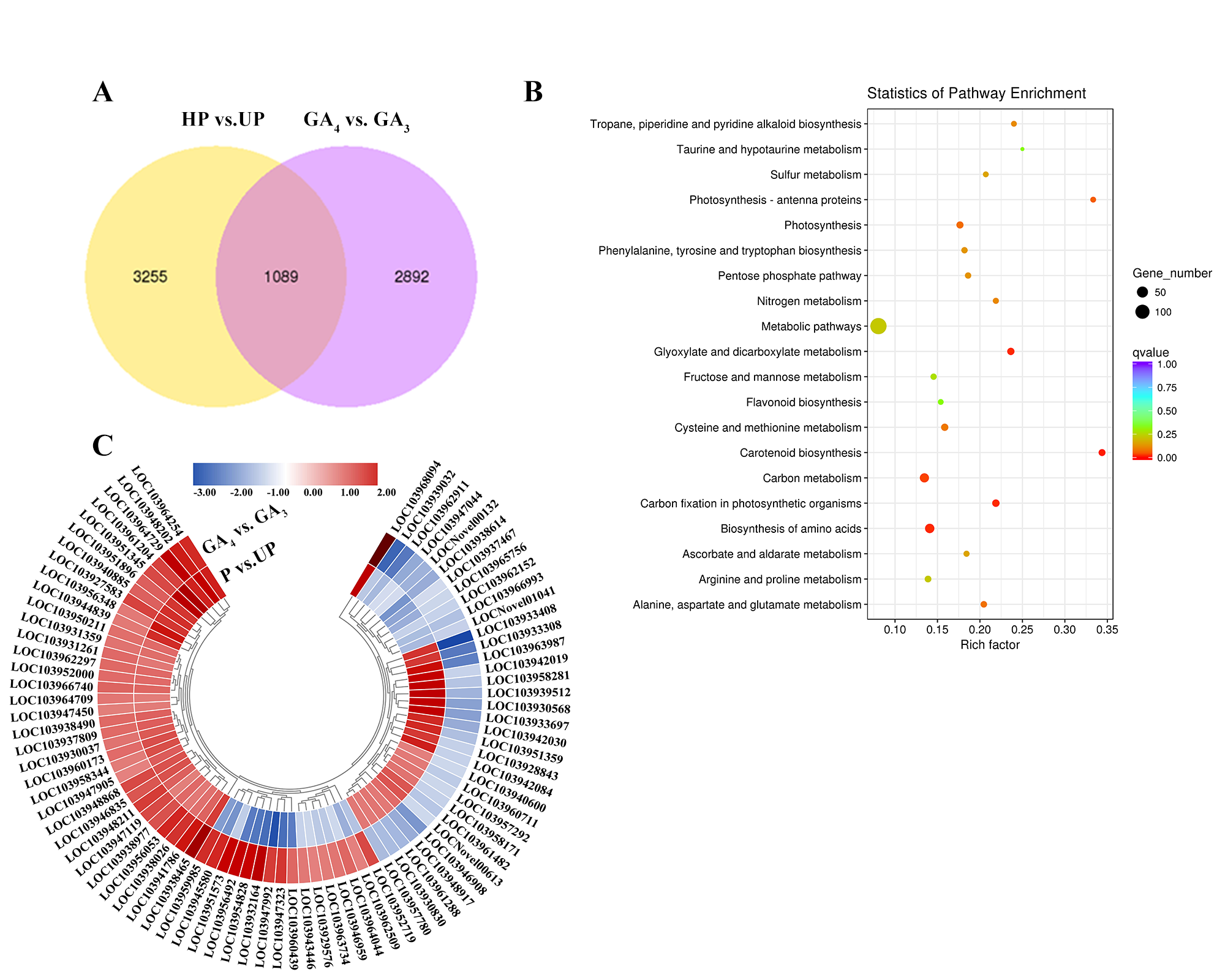

Supplement: Supplementary file 2 — Additional file 2: Figure Supplemental 2. Identification of PbCYP78A6 with transcription analysis (A) Number of differentially expressed genes between hand pollination treatment and un-pollination, GA4-treatment and GA3-treatment using venn diagram. (B) Top 20 pathways of KEGG functional enrichment among common DEGs between HP vs. UP and GA4 vs. GA3. (C) Relative expression of genes with the absolute value of fold change (|log2|>1). [file 12870_2021_3098_MOESM2_ESM.tif]

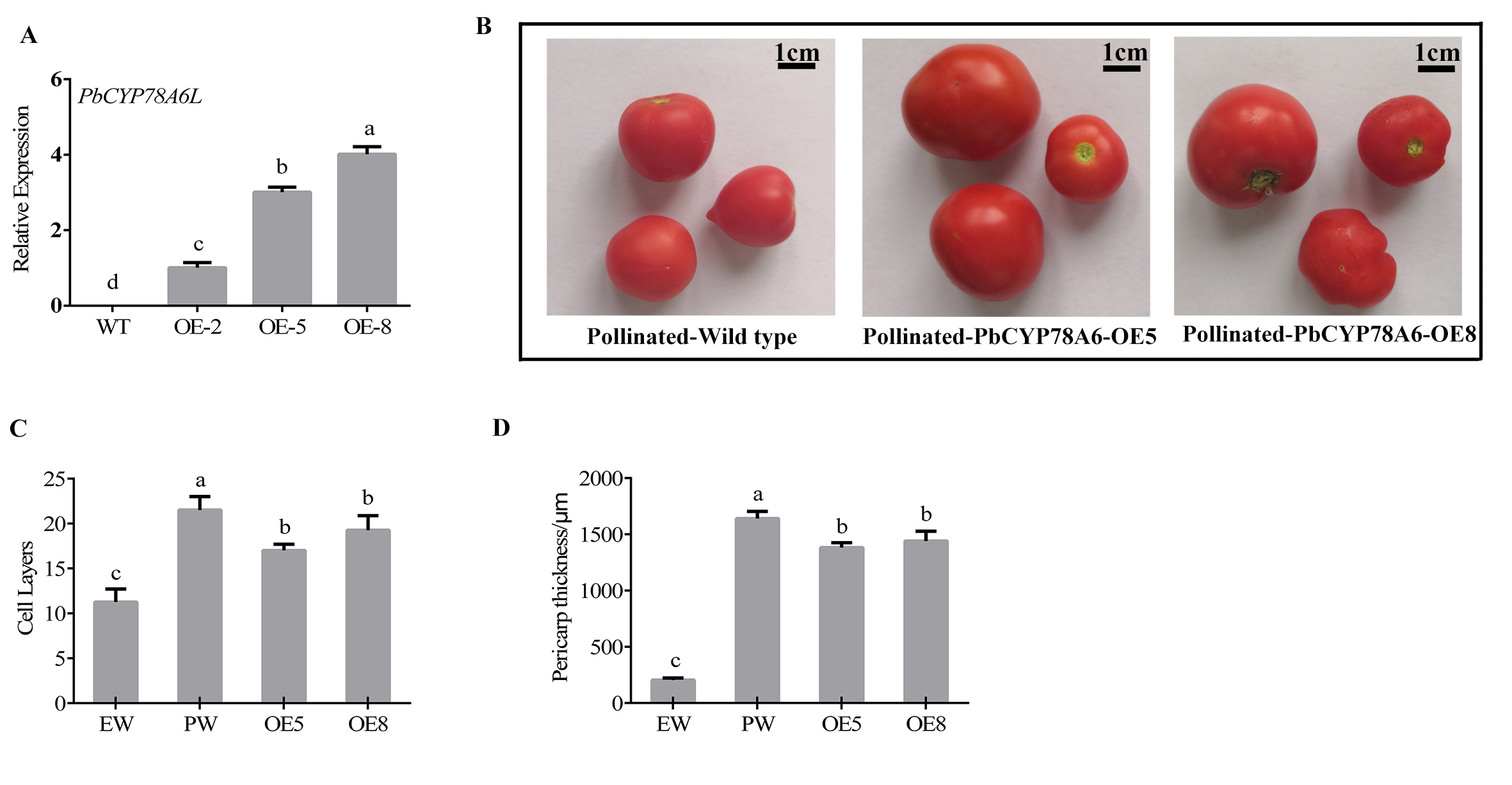

Supplement: Supplementary file 4 — Additional file 4: Figure Supplemental 3. Detection of the selected transgenic lines with overexpressing PbCYP78A6 gene and morphological, histological features of transgenic tomatoes. (A) The expression of PbCYP78A6 gene in transgenic tomatoes. (B) The morphological features of tomato lines with PbCYP78A6 overexpression and wild-type lines at the same trusses position under natural pollination. (C) The cell layers of transgenic tomatoes ovaries pericarp in PbCYP78A6 overexpression tomatoes. (D) The pericarp thickness of transgenic tomatoes ovaries in PbCYP78A6 overexpression tomatoes. EW, Emasculated Wild-type; PW, Pollinated Wild-type. The results represented are means of three biological replicates (±SD). Significant differences (P< 0.05) among treatments as determined by One-way ANOVA are indicated using different lowercase letters. [file 12870_2021_3098_MOESM4_ESM.tif]

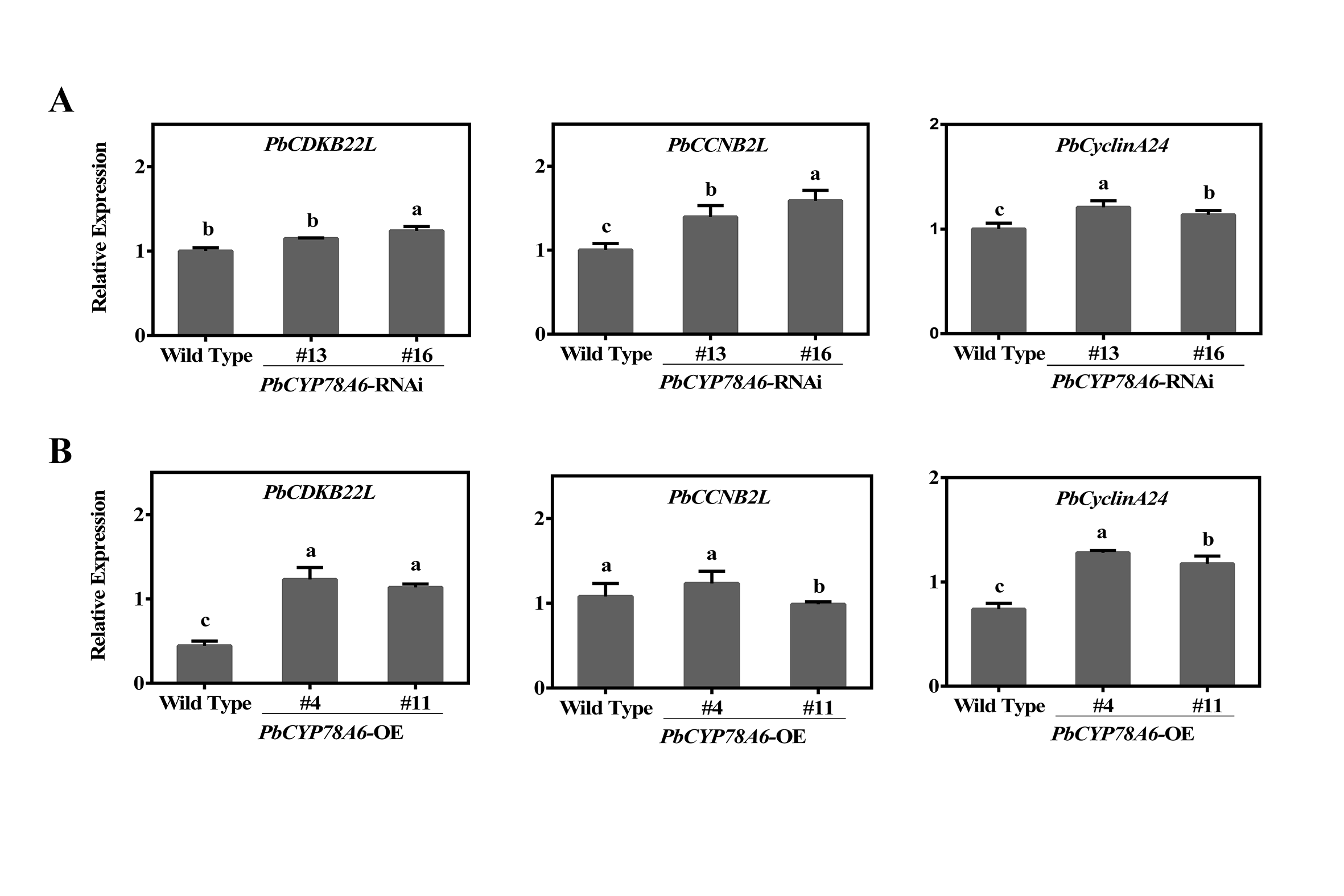

Supplement: Supplementary file 5 — Additional file 5: Figure Supplemental 4. The expression of cell division and expansion related genes in transgenic calli. A, RT-qPCR analysis of the expression levels of PbCDKB22L, PbCCNB2L, PbCyclinA24, in PbCYP78A6 RNAi pear calli. B, RT-qPCR analysis of the expression levels of PbCDKB22L, PbCCNB2L, PbCyclinA24 in PbCYP78A6 OE pear calli. The results represented are means of three biological replicates (±SD). Significant differences (P< 0.05) among treatments as determined by One-way ANOVA are indicated using different lowercase letters. [file 12870_2021_3098_MOESM5_ESM.tif]
